# Fast Approximations of the Jeffreys Divergence between Univariate Gaussian Mixtures via Mixture Conversions to Exponential-Polynomial Distributions

**DOI:** 10.3390/e23111417

**Published:** 2021-10-28

**Authors:** Frank Nielsen

**Affiliations:** Sony Computer Science Laboratories, Tokyo 141-0022, Japan; frank.nielsen.x@gmail.com

**Keywords:** Gaussian mixture model, Jeffreys divergence, mixture family, exponential-polynomial family, Maximum Likelihood Estimator, Score Matching Estimator, Hyvärinen divergence, relative Fisher information, moment matrix, Hankel matrix

## Abstract

The Jeffreys divergence is a renown arithmetic symmetrization of the oriented Kullback–Leibler divergence broadly used in information sciences. Since the Jeffreys divergence between Gaussian mixture models is not available in closed-form, various techniques with advantages and disadvantages have been proposed in the literature to either estimate, approximate, or lower and upper bound this divergence. In this paper, we propose a simple yet fast heuristic to approximate the Jeffreys divergence between two univariate Gaussian mixtures with arbitrary number of components. Our heuristic relies on converting the mixtures into pairs of dually parameterized probability densities belonging to an exponential-polynomial family. To measure with a closed-form formula the goodness of fit between a Gaussian mixture and an exponential-polynomial density approximating it, we generalize the Hyvärinen divergence to α-Hyvärinen divergences. In particular, the 2-Hyvärinen divergence allows us to perform model selection by choosing the order of the exponential-polynomial densities used to approximate the mixtures. We experimentally demonstrate that our heuristic to approximate the Jeffreys divergence between mixtures improves over the computational time of stochastic Monte Carlo estimations by several orders of magnitude while approximating the Jeffreys divergence reasonably well, especially when the mixtures have a very small number of modes.

## 1. Introduction

### 1.1. Statistical Mixtures and Statistical Divergences

We consider the problem of approximating the Jeffreys divergence [1] between two finite univariate continuous mixture models [2] $m\left(x\right)=\sum_{i=1}^{k}w_{i}p_{i}\left(x\right)$ and $m^{\prime }\left(x\right)=\sum_{i=1}^{k^{\prime }}w_{i}^{\prime }p_{i}^{\prime }\left(x\right)$ with continuous component distributions $p_{i}$’s and $p_{i}^{\prime \prime }$s defined on a coinciding support X⊂R. The mixtures m(x) and $m^{\prime }\left(x\right)$ may have a different number of components (i.e., $k\neq k^{\prime }$). Historically, Pearson [3] first considered a univariate Gaussian mixture of two components for modeling the distribution of the ratio of forehead breadth to body length of a thousand crabs in 1894 (Pearson obtained a unimodal mixture).

Although our work applies to any continuous mixtures of an exponential family (e.g., Rayleigh mixtures [4] with restricted support $X=R_{+}$), we explain our method for the most prominent family of mixtures encountered in practice: the Gaussian mixture models or GMMs for short. In the remainder, a univariate GMM $m\left(x\right)=\sum_{i=1}^{k}w_{i}p_{\mu_{i},\sigma_{i}}\left(x\right)$ with *k* Gaussian components

$$p_{i}\left(x\right)=p_{\mu_{i},\sigma_{i}}\left(x\right):=\frac{1}{\sigma_{i}\sqrt{2\pi }}\exp\left(-\frac{{\left(x-\mu_{i}\right)}^{2}}{2\sigma_{i}^{2}}\right),$$

is called a *k*-GMM.

The Kullback–Leibler divergence (KLD) [5,6] $D_{\mathrm{KL}}\left[m:m^{\prime }\right]$ between two mixtures *m* and $m^{\prime }$ is:

(1) $$D_{\mathrm{KL}}\left[m:m^{\prime }\right]:=\int_{X}m\left(x\right)\log\left(\frac{m(x)}{m^{\prime }\left(x\right)}\right)dx.$$

The KLD is an oriented divergence since $D_{\mathrm{KL}}\left[m:m^{\prime }\right]\neq D_{\mathrm{KL}}\left[m^{\prime }:m\right]$.

The Jeffreys divergence (JD) [1] $D_{J}\left[m,m^{\prime }\right]$ is the arithmetic symmetrization of the forward KLD and the reverse KLDs:

(2) $$\begin{array}{ccc} D_{J}\left[m,m^{\prime }\right] & := & D_{\mathrm{KL}}\left[m:m^{\prime }\right]+D_{\mathrm{KL}}\left[m^{\prime }:m\right], \end{array}$$

(3) $$\begin{array}{ccc} & = & \int_{X}\left(m\left(x\right)-m^{\prime }\left(x\right)\right)\log\left(\frac{m(x)}{m^{\prime }\left(x\right)}\right)dx. \end{array}$$

The JD is a symmetric divergence: $D_{J}\left[m,m^{\prime }\right]=D_{J}\left[m^{\prime },m\right]$. In the literature, the Jeffreys divergence [7] has also been called the *J*-divergence [8,9], the symmetric Kullback–Leibler divergence [10] and sometimes the symmetrical Kullback–Leibler divergence [11,12]. In general, it is provably hard to calculate the definite integral of the KLD between two continuous mixtures in closed-form: For example, the KLD between two GMMs has been shown to be non-analytic [13]. Thus, in practice, when calculating the JD between two GMMs, one can either approximate [14,15], estimate [16], or bound [17,18] the KLD between mixtures. Another approach to bypass the computational intractability of calculating the KLD between mixtures consists of designing new types of divergences that admit closed-form expressions for mixtures. See, for example, the Cauchy–Schwarz divergence [19] or the total square divergence [20] (a total Bregman divergence) that admit the closed-form formula when handling GMMs. The total square divergence [20] is invariant to rigid transformations and provably robust to outliers in clustering applications.

In practice, to estimate the KLD between mixtures, one uses the following Monte Carlo (MC) estimator:

$${\hat{D}}_{\mathrm{KL}}^{S_{s}}\left[m:m^{\prime }\right]:=\frac{1}{s}\sum_{i=1}^{s}\left(\log\left(\frac{m(x_{i})}{m^{\prime }\left(x_{i}\right)}\right)+\frac{m^{\prime }\left(x_{i}\right)}{m(x_{i})}-1\right)\geq 0,$$

where $S_{s}=\left\{x_{1},\ldots ,x_{s}\right\}$ is *s* independent and identically distributed (i.i.d.) samples from m(x). This MC estimator is by construction always non-negative and therefore consistent. That is, we have $\lim_{s\to \infty }{\hat{D}}_{\mathrm{KL}}^{S_{s}}\left[m:m^{\prime }\right]=D_{\mathrm{KL}}\left[m:m^{\prime }\right]$ under mild conditions [21].

Similarly, we estimate the Jeffreys divergence via MC sampling as follows:

(4) $${\hat{D}}_{J}^{S_{s}}\left[m,m^{\prime }\right]:=\frac{1}{s}\sum_{i=1}^{s}2\frac{\left(m\left(x_{i}\right)-m^{\prime }\left(x_{i}\right)\right)}{m\left(x_{i}\right)+m^{\prime }\left(x_{i}\right)}\log\left(\frac{m(x_{i})}{m^{\prime }\left(x_{i}\right)}\right)\geq 0,$$

where $S_{s}=\left\{x_{1},\ldots ,x_{s}\right\}$ are *s* i.i.d. samples from the “middle mixture” $m_{12}\left(x\right):=\frac{1}{2}\left(m\left(x\right)+m^{\prime }\left(x\right)\right)$. By choosing the middle mixture $m_{12}\left(x\right)$ for sampling, we ensure that we keep the symmetric property of the JD (i.e., ${\hat{D}}_{J}^{S_{s}}\left[m,m^{\prime }\right]={\hat{D}}_{J}^{S_{s}}\left[m^{\prime },m\right]$), and we also have consistency under mild conditions [21]: $\lim_{s\to \infty }{\hat{D}}_{J}^{S_{s}}\left[m,m^{\prime }\right]=D_{J}\left[m,m^{\prime }\right]$. The time complexity to stochastically estimate the JD is $\tilde{O}\left(\left(k+k^{\prime }\right)s\right)$, with *s* typically ranging from $10^{4}$ to $10^{6}$ in applications. Notice that the number of components of a mixture can be very large (e.g., k=O(n) for *n* input data when using Kernel Density Estimators [2]). KDEs may also have a large number of components and may potentially exhibit many spurious modes visualized as small bumps when plotting the densities.

### 1.2. Jeffreys Divergence between Densities of an Exponential Family

We consider approximating the JD by converting continuous mixtures into densities of exponential families [22]. A continuous exponential family (EF) $E_{t}$ of order *D* is defined as a family of probability density functions with support X and the probability density function:

$$E_{t}:=\left\{p_{\theta }\left(x\right):=\exp\left(\sum_{i=1}^{D}\theta_{i}t_{i}\left(x\right)-F\left(\theta \right)\right)\;:\;\theta \in \Theta \right\},$$

where F(θ) is called the log-normalizer, which ensures the normalization of $p_{\theta }\left(x\right)$ (i.e., $\int_{X}p_{\theta }\left(x\right)dx=1$):

$$F\left(\theta \right)=\log\left(\int_{X}\exp\left(\sum_{i=1}^{D}\theta_{i}t_{i}\left(x\right)\right)dx\right).$$

Parameter $\theta \in \Theta \subset R^{D}$ is called the natural parameter, and the functions $t_{1}\left(x\right)$, *…*, $t_{D}\left(x\right)$ are called the sufficient statistics [22]. Let Θ denote the natural parameter space: Θ:={θ:F(θ)<∞}, an open convex domain for regular exponential families [22]. The exponential family is said to be minimal when the functions $1,t_{1}\left(x\right),\ldots ,t_{D}\left(x\right)$ are linearly independent.

It is well-known that one can bypass the definite integral calculation of the KLD when the probability density functions $p_{\theta }$ and $p_{\theta^{\prime }}$ belong to the same exponential family [23,24]:

$$D_{\mathrm{KL}}\left[p_{\theta }:p_{\theta }\right]=B_{F}\left(\theta^{\prime }:\theta \right),$$

where $B_{F}\left(\theta_{2}:\theta_{1}\right)$ is the Bregman divergence induced by the log-normalizer, a strictly convex real-analytic function [22]. The Bregman divergence [25] between two parameters $\theta_{1}$ and $\theta_{2}$ for a strictly convex and smooth generator *F* is defined by:

(5) $$B_{F}\left(\theta_{1}:\theta_{2}\right):=F\left(\theta_{1}\right)-F\left(\theta_{2}\right)-{\left(\theta_{1}-\theta_{2}\right)}^{⊤}\nabla F\left(\theta_{2}\right).$$

Thus, the Jeffreys divergence between two pdfs $p_{\theta }$ and $p_{\theta^{\prime }}$ belonging to the same exponential family is a symmetrized Bregman divergence [26]:

$$\begin{array}{ccc} D_{J}\left[p_{\theta }:p_{\theta }\right] & = & B_{F}\left(\theta^{\prime }:\theta \right)+B_{F}\left(\theta :\theta^{\prime }\right),\\ & = & {\left(\theta^{\prime }-\theta \right)}^{⊤}\left(\nabla F\left(\theta^{\prime }\right)-\nabla F\left(\theta \right)\right). \end{array}$$

Let $F^{*}\left(\eta \right)$ denote the Legendre–Fenchel convex conjugate of F(θ):

(6) $$F^{*}\left(\eta \right):=\underset{\theta \in \Theta }{\sup}\left\{\theta^{⊤}\eta -F\left(\theta \right)\right\}.$$

The Legendre transform ensures that η=∇F(θ) and $\theta =\nabla F^{*}\left(\eta \right)$, and the Jeffreys divergence between two pdfs $p_{\theta }$ and $p_{\theta^{\prime }}$ belonging to the same exponential family is:

(7) $$D_{J}\left[p_{\theta }:p_{\theta }\right]={\left(\theta^{\prime }-\theta \right)}^{⊤}\left(\eta^{\prime }-\eta \right).$$

Notice that the log-normalizer F(θ) does not appear explicitly in the above formula.

### 1.3. A Simple Approximation Heuristic

Densities $p_{\theta }$ of an exponential family admit a dual parameterization [22]: $\eta =\eta \left(\theta \right):=E_{p_{\theta }}\left[t\left(x\right)\right]=\nabla F\left(\theta \right)$, called the moment parameterization (or mean parameterization). Let *H* denote the moment parameter space. Let us use the subscript and superscript notations to emphasize the coordinate system used to index a density: In our notation, we thus write $p_{\theta }\left(x\right)=p^{\eta }\left(x\right)$.

In view of Equation (7), our method to approximate the Jeffreys divergence between mixtures *m* and $m^{\prime }$ consists of first converting those mixtures *m* and $m^{\prime }$ into pairs of polynomial exponential densities (PEDs) in Section 2. To convert a mixture m(x) into a pair $\left(p_{{\bar{\theta }}_{1}},p^{{\bar{\eta }}_{2}}\right)$ dually parameterized (but not dual because ${\bar{\eta }}_{2}\neq \nabla F\left({\bar{\theta }}_{1}\right)$), we shall consider “integral extensions” (or information projections) of the Maximum Likelihood Estimator [22] (MLE estimates in the moment parameter space H={∇F(θ):θ∈Θ}) and of the Score Matching Estimator [27] (SME estimates in the natural parameter space $\Theta =\{\nabla F^{*}\left(\eta \right)\;:\;\eta \in H\}$).

**Figure 1:**
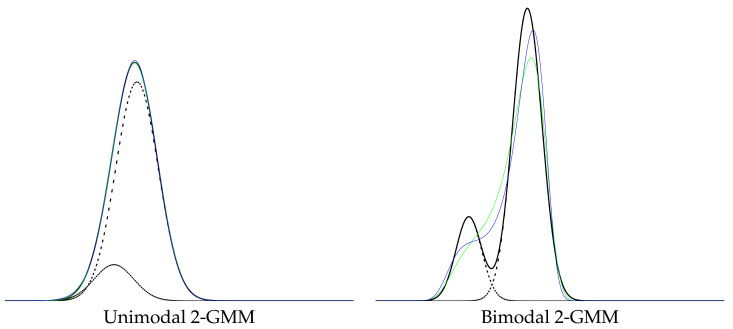
Two examples illustrating the conversion of a GMM *m* (black) of k=2 components (dashed black) into a pair of polynomial exponential densities of order D=4$\left(p_{{\bar{\theta }}_{\mathrm{SME}}},p^{{\bar{\eta }}_{\mathrm{MLE}}}\right)$. PED $p_{{\bar{\theta }}_{\mathrm{SME}}}$ is displayed in green, and PED $p^{{\bar{\eta }}_{\mathrm{MLE}}}$ is displayed in blue. To display $p^{{\bar{\eta }}_{\mathrm{MLE}}}$, we first converted ${\bar{\eta }}_{\mathrm{MLE}}$ to ${\tilde{\bar{\theta }}}_{\mathrm{MLE}}$ using an iterative linear system descent method (ILSDM), and we numerically estimated the normalizing factors $Z({\bar{\theta }}_{\mathrm{SME}})$ and $Z({\bar{\eta }}_{\mathrm{MLE}})$ to display the normalized PEDs.

We shall consider polynomial exponential families [28] (PEFs) also called exponential-polynomial families (EPFs) [29]. PEFs $E_{D}$ are regular minimal exponential families with polynomial sufficient statistics $t_{i}\left(x\right)=x^{i}$ for i∈{1,…,D}. For example, the exponential distributions $\left\{p_{\lambda }\left(x\right)=\lambda \exp\left(-\lambda x\right)\right\}$ form a PEF with D=1, t(x)=x and $X=R_{+}$, and the normal distributions form an EPF with D=2, $t\left(x\right)={\left[x\;x^{2}\right]}^{⊤}$ and X=R, etc. Although the log-normalizer F(θ) can be obtained in closed-form for lower order PEFs (e.g., D=1 or D=2) or very special subfamilies (e.g., when D=1 and $t_{1}\left(x\right)=x^{k}$, exponential-monomial families [30]), a no-closed form formula is available for F(θ) of EPFs in general as soon D≥4 [31,32], and the cumulant function F(θ) is said to be computationally intractable. Notice that when X=R, the leading coefficient $\theta_{D}$ is negative for even integer order *D*. EPFs are attractive because these families can universally model any smooth multimodal distribution [28] and require fewer parameters in comparison to GMMs: Indeed, a univariate *k*-GMM m(x) (at most *k* modes and k−1 antimodes) requires 3k−1 parameters to specify m(x) (or k+1 for a KDE with constant kernel width σ or 2k−1 for a KDE with varying kernel widths, but then k=n observations). A density of an EPF of order *D* is called an exponential-polynomial density (EPD) and requires *D* parameters to specify θ, with, at most, $\frac{D}{2}$ modes (and $\frac{D}{2}-1$ antimodes). The case of the quartic (polynomial) exponential densities $E_{4}$ (D=4) has been extensively investigated in [31,33,34,35,36,37]. Armstrong and Brigo [38] discussed order-6 PEDs, and Efron and Hastie reported and order-7 PEF in their textbook (see Figure 5.7 of [39]). Figure 1 displays two examples of converting a GMM into a pair of dually parameterized exponential-polynomial densities.

Then by converting both mixture *m* and mixture $m^{\prime }$ into pairs of dually natural/moment parameterized unnormalized PEDs, i.e., $m\to (q_{{\bar{\theta }}_{\mathrm{SME}}},q_{{\bar{\eta }}_{\mathrm{MLE}}})$ and $m^{\prime }\to \left(q_{{\bar{\theta }}_{\mathrm{SME}}^{\prime }},q_{{\bar{\eta }}_{\mathrm{MLE}}}^{\prime }\right)$, we approximate the JD between mixtures *m* and $m^{\prime }$ by using the four parameters of the PEDs

(8) $$D_{J}\left[m,m^{\prime }\right]\approx {\left({\bar{\theta }}_{\mathrm{SME}}^{\prime }-{\bar{\theta }}_{\mathrm{SME}}\right)}^{⊤}\left({\bar{\eta }}_{\mathrm{MLE}}^{\prime }-{\bar{\eta }}_{\mathrm{MLE}}\right).$$

Let $\Delta_{J}$ denote the approximation formula obtained from the two pairs of PEDs:

(9) $$\Delta_{J}\left[p_{\theta_{\mathrm{SME}}},p^{\eta_{\mathrm{MLE}}};p_{\theta_{\mathrm{SME}}^{\prime }},p^{\eta_{\mathrm{MLE}}^{\prime }}\right]:={\left(\theta_{\mathrm{SME}}^{\prime }-\theta_{\mathrm{SME}}\right)}^{⊤}\left(\eta_{\mathrm{MLE}}^{\prime }-\eta_{\mathrm{MLE}}\right).$$

Let $\Delta_{J}\left(\theta_{\mathrm{SME}},\eta_{\mathrm{MLE}};\theta_{\mathrm{SME}}^{\prime },\eta_{\mathrm{MLE}}^{\prime }\right):=\Delta_{J}\left[p_{\theta_{\mathrm{SME}}},p^{\eta_{\mathrm{MLE}}};p_{\theta_{\mathrm{SME}}^{\prime }},p^{\eta_{\mathrm{MLE}}^{\prime }}\right]$. Then we have

$$D_{J}\left[m,m^{\prime }\right]\approx {\tilde{D}}_{J}\left[m,m^{\prime }\right]:=\Delta_{J}\left(\theta_{\mathrm{SME}},\eta_{\mathrm{MLE}};\theta_{\mathrm{SME}}^{\prime },\eta_{\mathrm{MLE}}^{\prime }\right).$$

Note that $\Delta_{J}$ is not a proper divergence as it may be negative since, in general, ${\bar{\eta }}_{\mathrm{MLE}}\neq \nabla F\left({\bar{\theta }}_{\mathrm{SME}}\right)$. That is, $\Delta_{J}$ may not satisfy the law of the indiscernibles. Approximation $\Delta_{J}$ is exact when $k_{1}=k_{2}=1$, with both *m* and $m^{\prime }$ belonging to an exponential family.

We experimentally show in Section 4 that the ${\tilde{D}}_{J}$ heuristic yields fast approximations of the JD compared to the MC baseline estimations by several order of magnitudes while approximating the JD reasonably well when the mixtures have a small number of modes.

**Figure 2:**
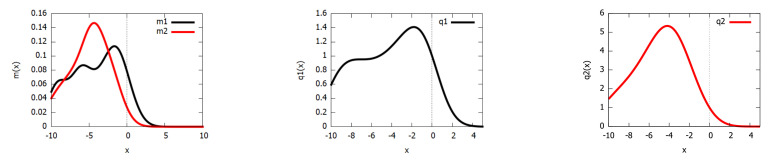
Two mixtures $m_{1}$ (black) and $m_{2}$ (red) of $k_{1}=10$ components and $k_{2}=11$ components (**left**), respectively. The unnormalized PEFs $q_{{\bar{\theta }}_{1}}={\tilde{p}}_{{\bar{\theta }}_{1}}$ (**middle**) and $q_{{\bar{\theta }}_{2}}={\tilde{p}}_{{\bar{\theta }}_{2}}$ (**right**) of order D=8. Jeffreys divergence (about 0.2634) is approximated using PEDs within 0.6% compared to the Monte Carlo estimate with a speed factor of about 3190. Notice that displaying $p_{{\bar{\theta }}_{1}}$ and $p_{{\bar{\theta }}_{2}}$ on the same PDF canvas as the mixtures would require calculating the partition functions $Z({\bar{\theta }}_{1})$ and $Z({\bar{\theta }}_{2})$ (which we do not in this figure). The PEDs $q^{{\bar{\eta }}_{1}}$ and $q^{{\bar{\eta }}_{2}}$ of the pairs $\left({\bar{\theta }}_{1},{\bar{\eta }}_{1}\right)$ and $\left({\bar{\theta }}_{2},{\bar{\eta }}_{2}\right)$ parameterized in the moment space are not shown here.

For example, Figure 2 displays the unnormalized PEDs obtained for two Gaussian mixture models ($k_{1}=10$ components and $k_{2}=11$ components) into PEDs of a PEF of order D=8. The MC estimation of the JD with $s=10^{6}$ samples yields 0.2633⋯, while the PED approximation of Equation (8) on corresponding PEFs yields 0.2618… (the relative error is 0.00585⋯ or about 0.585…%). It took about 2642.581 milliseconds (with $s=10^{6}$ on a Dell Inspiron 7472 laptop) to MC estimate the JD, while it took about 0.827 milliseconds with the PEF approximation. Thus, we obtained a speed-up factor of about 3190 (three orders of magnitude) for this particular example. Notice that when viewing Figure 2, we tend to visually evaluate the dissimilarity using the total variation distance (a metric distance):

$$D_{\mathrm{TV}}\left[m,m^{\prime }\right]:=\frac{1}{2}\int \left|m\left(x\right)-m^{\prime }\left(x\right)\right|dx,$$

rather than by a dissimilarity relating to the KLD. Using Pinsker’s inequality [40,41], we have $D_{J}\left[m,m^{\prime }\right]\geq D_{\mathrm{TV}}{\left[m,m^{\prime }\right]}^{2}$ and $D_{\mathrm{TV}}\left[m,m^{\prime }\right]\in \left[0,1\right]$. Thus, large TV distance (e.g., $D_{\mathrm{TV}}\left[m,m^{\prime }\right]=0.1$) between mixtures may have a small JD since Pinsker’s inequality yields $D_{J}\left[m,m^{\prime }\right]\geq 0.01$.

Let us point out that our approximation heuristic is deterministic, while the MC estimations are stochastic: That is, each MC run (Equation (4)) returns a different result, and a single MC run may yield a very bad approximation of the true Jeffreys divergence.

We compare our fast heuristic ${\tilde{D}}_{J}\left[m,m^{\prime }\right]={\left(\theta_{\mathrm{SME}}^{\prime }-\theta_{\mathrm{SME}}\right)}^{⊤}\left(\eta_{\mathrm{MLE}}^{\prime }-\eta_{\mathrm{MLE}}\right)$ with two more costly methods relying on numerical procedures to convert natural ↔ moment parameters:

1. Simplify GMMs $m_{i}$ into $p^{\eta_{i}^{\mathrm{MLE}}}$, and approximately convert the ${\bar{\eta }}_{i}^{\mathrm{MLE}}$’s into ${\tilde{\theta }}_{i}^{\mathrm{MLE}}$’s. Then approximate the Jeffreys divergence as
  (10) $$D_{J}\left[m_{1},m_{2}\right]≃{\tilde{\Delta }}_{J}^{\mathrm{MLE}}\left[m_{1},m_{2}\right]:={\left({\tilde{\theta }}_{2}^{\mathrm{MLE}}-{\tilde{\theta }}_{1}^{\mathrm{MLE}}\right)}^{⊤}\left({\bar{\eta }}_{2}^{\mathrm{MLE}}-{\bar{\eta }}_{1}^{\mathrm{MLE}}\right).$$
2. Simplify GMMs $m_{i}$ into $p_{{\bar{\theta }}_{i}^{\mathrm{SME}}}$, and approximately convert the ${\bar{\theta }}_{i}^{\mathrm{SME}}$’s into ${\tilde{\eta }}_{i}^{\mathrm{SME}}$’s. Then approximate the Jeffreys divergence as
  (11) $$D_{J}\left[m_{1},m_{2}\right]≃{\tilde{\Delta }}_{J}^{\mathrm{SME}}\left(m_{1},m_{2}\right)={\left({\bar{\theta }}_{2}^{\mathrm{SME}}-{\bar{\theta }}_{1}^{\mathrm{SME}}\right)}^{⊤}\left({\tilde{\eta }}_{2}^{\mathrm{SME}}-{\tilde{\eta }}_{1}^{\mathrm{SME}}\right).$$

### 1.4. Contributions and Paper Outline

Our contributions are summarized as follows:

- We explain how to convert any continuous density r(x) (including GMMs) into a polynomial exponential density in Section 2 using integral-based extensions of the Maximum Likelihood Estimator [22] (MLE estimates in the moment parameter space *H*, Theorem 1 and Corollary 1) and the Score Matching Estimator [27] (SME estimates in the natural parameter space Θ, Theorem 3). We show a connection between SME and the Moment Linear System Estimator [28] (MLSE).
- We report a closed-form formula to evaluate the goodness-of-fit of a polynomial family density to a GMM in Section 3 using an extension of the Hyvärinen divergence [42] (Theorem 4) and discuss the problem of model selection for choosing the order *D* of the polynomial exponential family.
- We show how to approximate the Jeffreys divergence between GMMs using a pair of natural/moment parameter PED conversion and present experimental results that display a gain of several orders of magnitude of performance when compared to the vanilla Monte Carlo estimator in Section 4. We observe that the quality of the approximations depend on the number of modes of the GMMs [43]. However, calculating or counting the modes of a GMM is a difficult problem in its own [43].

The paper is organized as follows: In Section 2, we show how to convert arbitrary probability density functions into polynomial exponential densities using the integral-based Maximum Likelihood Estimator (MLE) and Score Matching Estimator (SME). We describe a Maximum Entropy method to iteratively convert moment parameters into natural parameters in Section 2.3.1. It is followed by Section 3, which shows how to calculate in closed-form the order-2 Hyvärinen divergence between a GMM and a polynomial exponential density. We use this criterion to perform model selection. Section 4 presents our computational experiments that demonstrate a gain of several orders of magnitudes for GMMs with a small number of modes. Finally, we conclude in Section 5.

## 2. Converting Finite Mixtures to Exponential Family Densities

We report two generic methods to convert a mixture m(x) into a density $p_{\theta }\left(x\right)$ of an exponential family: The first method extending the MLE in Section 2.1 proceeds using the mean parameterization η, while the second method extending the SME in Section 2.2 uses the natural parameterization of the exponential family. We then describe how to convert the moments parameters into natural parameters (and vice versa) for polynomial exponential families in Section 2.3. We show how to instantiate these generic conversion methods for GMMs: It requires calculating non-central moments of GMMs in closed-form. The efficient computations of raw moments of GMMs is detailed in Section 2.4.

### 2.1. Conversion Using the Moment Parameterization (MLE)

Let us recall that in order to estimate the moment or mean parameter ${\hat{\eta }}_{\mathrm{MLE}}$ of a density belonging an exponential family

$$E_{t}:=\left\{p_{\theta }\left(x\right)=\exp\left(t{\left(x\right)}^{⊤}\theta -F\left(\theta \right)\right)\right\}$$

with a sufficient statistic vector $t\left(x\right)={\left[t_{1}\left(x\right)\;\ldots \;t_{D}\left(x\right)\right]}^{⊤}$ from an i.i.d. sample set $x_{1},\ldots ,x_{n}$, the Maximum Likelihood Estimator (MLE) [22,44] yields

(12) $$\begin{array}{ccc} & & \max_{\theta }\prod_{i=1}^{n}p_{\theta }\left(x_{i}\right), \end{array}$$

(13) $$\begin{array}{ccc} & \equiv & \max_{\theta }\sum_{i=1}^{n}\log p_{\theta }\left(x_{i}\right), \end{array}$$

(14) $$\begin{array}{ccc} & = & \max_{\theta }E\left(\theta \right):=\left(\sum_{i=1}^{n}t{\left(x_{i}\right)}^{⊤}\theta \right)-nF\left(\theta \right), \end{array}$$

(15) $$\begin{array}{ccc} & \Rightarrow & {\hat{\eta }}_{\mathrm{MLE}}=\frac{1}{n}\sum_{i=1}^{n}t\left(x_{i}\right). \end{array}$$

In statistics, Equation (12) is called the estimating equation. The MLE exists under mild conditions [22] and is unique since the Hessian $\nabla^{2}E\left(\theta \right)=\nabla^{2}F\left(\theta \right)$ of the estimating equation is positive-definite (log-normalizers F(θ) are always strictly convex and real analytic [22]). The MLE is consistent and asymptotically normally distributed [22]. Furthermore, since the MLE satisfies the equivariance property [22], we have ${\hat{\theta }}_{\mathrm{MLE}}=\nabla F^{*}\left({\hat{\eta }}_{\mathrm{MLE}}\right)$, where $\nabla F^{*}$ denotes the gradient of the conjugate function $F^{*}\left(\eta \right)$ of the cumulant function F(θ) of the exponential family. In general, $\nabla F^{*}$ is intractable for PEDs with D≥4.

By considering the empirical distribution

$$p_{e}\left(x\right):=\frac{1}{n}\sum_{i=1}^{s}\delta_{x_{i}}\left(x\right),$$

where $\delta_{x_{i}}\left(\cdot \right)$ denotes the Dirac distribution at location $x_{i}$, we can formulate the MLE problem as a minimum KLD problem between the empirical distribution and a density of the exponential family:

$$\begin{array}{ccc} \min_{\theta }D_{\mathrm{KL}}\left[p_{e}:p_{\theta }\right] & = & \min-H\left[p_{e}\right]-E_{p_{e}}\left[\log p_{\theta }\left(x\right)\right],\\ & \equiv & \max_{\theta }\frac{1}{n}\sum_{i=1}^{n}\log p_{\theta }\left(x_{i}\right), \end{array}$$

since the entropy term $H[p_{e}]$ is independent of θ.

Thus, to convert an arbitrary smooth density r(x) into a density $p_{\theta }$ of an exponential family $E_{t}$, we have to solve the following minimization problem:

$$\min_{\theta \in \Theta }D_{\mathrm{KL}}\left[r:p_{\theta }\right].$$

Rewriting the minimization problem as:

$$\begin{array}{ccc} & & \min_{\theta }D_{\mathrm{KL}}\left[r:p_{\theta }\right]=-\int r\left(x\right)\log p_{\theta }\left(x\right)dx+\int r\left(x\right)\log r\left(x\right)dx,\\ & \equiv & \min_{\theta }-\int r\left(x\right)\log p_{\theta }\left(x\right)dx,\\ & = & \min_{\theta }\int r\left(x\right)\left(F\left(\theta \right)-\theta^{⊤}t\left(x\right)\right)dx,\\ & = & \min_{\theta }\bar{E}\left(\theta \right)=F\left(\theta \right)-\theta^{⊤}E_{r}\left[t\left(x\right)\right], \end{array}$$

we obtain

(16) $${\bar{\eta }}_{\mathrm{MLE}}\left(r\right):=E_{r}\left[t\left(x\right)\right]=\int_{X}r\left(x\right)t\left(x\right)dx.$$

The minimum is unique since $\nabla^{2}\bar{E}\left(\theta \right)=\nabla^{2}F\left(\theta \right)≻0$ (positive-definite matrix). This conversion procedure $r\left(x\right)\to p^{{\bar{\eta }}_{\mathrm{MLE}}\left(r\right)}\left(x\right)$ can be interpreted as an integral extension of the MLE, hence the $\bar{\dot{}}$ notation in ${\bar{\eta }}_{\mathrm{MLE}}$. Notice that the ordinary MLE is ${\hat{\eta }}_{\mathrm{MLE}}={\bar{\eta }}_{\mathrm{MLE}}\left(p_{e}\right)$ obtained for the empirical distribution: $r=p_{e}$: ${\bar{\eta }}_{\mathrm{MLE}}\left(p_{e}\right)=\frac{1}{n}\sum_{i=1}^{n}t\left(x_{i}\right)$.

**Theorem** **1.** *The best density $p^{\bar{\eta }}\left(x\right)$ of an exponential family $E_{t}=\left\{p_{\theta }\;:\;\theta \in \Theta \right\}$ minimizing the Kullback–Leibler divergence $D_{\mathrm{KL}}\left[r:p_{\theta }\right]$ between a density r and a density $p_{\theta }$ of an exponential family $E_{t}$ is $\bar{\eta }=E_{r}\left[t\left(x\right)\right]=\int_{X}r\left(x\right)t\left(x\right)dx$.*

Notice that when $r=p_{\theta }$, we obtain $\bar{\eta }=E_{p_{\theta }}\left[t\left(x\right)\right]=\eta$ so that the method ${\bar{\eta }}_{\mathrm{MLE}}\left(r\right)$ is consistent (by analogy to the finite i.i.d. MLE case): ${\bar{\eta }}_{\mathrm{MLE}}\left(p_{\theta }\right)=\eta =\nabla F\left(\theta \right)$.

The KLD right-sided minimization problem can be interpreted as an information projection of *r* onto $E_{t}$. As a corollary of Theorem 1, we obtain:

**Corollary** **1** (Best right-sided KLD simplification of a mixture). *The best right-sided KLD simplification of a homogeneous mixture of exponential families [2] $m\left(x\right)=\sum_{i=1}^{k}w_{i}p_{\theta_{i}}\left(x\right)$ with $p_{\theta_{i}}\in E_{t}$, i.e., $\min_{\theta \in \Theta }D_{\mathrm{KL}}\left[m:p_{\theta }\right]$, into a single component $p^{\eta }\left(x\right)$ is given by $\eta ={\hat{\eta }}_{\mathrm{MLE}}\left(m\right)=E_{m}\left[t\left(x\right)\right]=\sum_{i=1}^{k}\eta_{i}=\bar{\eta }$.*

**Figure 3:**
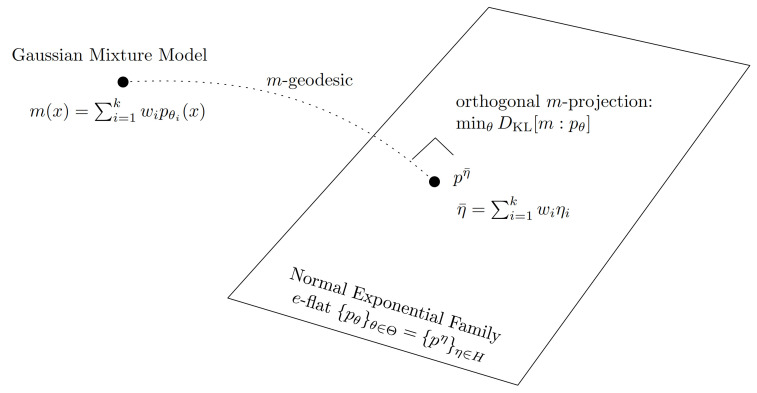
The best simplification of a GMM m(x) into a single normal component $p_{\theta^{*}}$ ($\min_{\theta \in \Theta }D_{\mathrm{KL}}\left[m:p_{\theta }\right]=\min_{\eta \in H}D_{\mathrm{KL}}\left[m:p^{\eta }\right]$) is geometrically interpreted as the unique *m*-projection of m(x) onto the Gaussian family (a *e*-flat): We have $\eta^{*}=\bar{\eta }=\sum_{i=1}^{k}\eta_{i}$.

Equation (16) allows us to greatly simplify the proofs reported in [45] for mixture simplifications that involved the explicit use of the Pythagoras’ theorem in the dually flat spaces of exponential families [42]. Figure 3 displays the geometric interpretation of the best KLD simplification of a GMM with ambient space the probability space $\left(R,B\left(R\right),\mu_{L}\right)$, where $\mu_{L}$ denotes the Lebesgue measure and B(R) the Borel σ-algebra of R.

Let us notice that Theorem 1 yields an algebraic system for polynomial exponential densities, i.e., $E_{m}\left[x^{i}\right]={\bar{\eta }}_{i}$ for i∈{1,…,D}, to compute ${\bar{\eta }}_{\mathrm{MLE}}\left(m\right)$ for a given GMM m(x) (since raw moments $E_{m}\left[x^{i}\right]$ are algebraic). In contrast with this result, the MLE of i.i.d. observations is in general not an algebraic function [46] but a transcendental function.

### 2.2. Converting to a PEF Using the Natural Parameterization (SME)

#### Integral-Based Score Matching Estimator (SME)

To convert the density r(x) into an exponential density with sufficient statistics t(x), we can also use the Score Matching Estimator [27,47] (SME). The Score Matching Estimator minimizes the Hyvärinen divergence $D_{H}$ (Equation (4) of [47]):

$$D_{H}\left[p:p_{\theta }\right]:=\frac{1}{2}\int {∥\nabla_{x}\log p\left(x\right)-\nabla_{x}\log p_{\theta }\left(x\right)∥}^{2}\;p\left(x\right)dx.$$

The Hyvärinen divergence is also known as half of the relative Fisher information in the optimal transport community (Equation (8) of [48] or Equation (2.2) in [49]), where it is defined for two measures μ and ν as follows:

$$I\left[\mu :\nu \right]:=\int_{X}{∥\nabla \log\frac{d\mu }{d\nu }∥}^{2}d\mu =4\int_{X}{∥\nabla \sqrt{\frac{d\mu }{d\nu }}∥}^{2}d\nu .$$

Moreover, the relative Fisher information can be defined on complete Riemannian manifolds [48].

That is, we convert a density r(x) into an exponential family density $p_{\theta }\left(x\right)$ using the following minimizing problem:

$$\theta_{\mathrm{SME}}\left(r\right)=\min_{\theta \in \Theta }D_{H}\left[r:p_{\theta }\right].$$

Beware that in statistics, the score $s_{\theta }\left(x\right)$ is defined by $\nabla_{\theta }\log p_{\theta }\left(x\right)$, but in Score Matching, we refer to the “data score” defined by $\nabla_{x}\log p_{\theta }\left(x\right)$. Hyvärinen [47] gave an explanation of the naming “score” using a spurious location parameter.

- Generic solution: It can be shown that for exponential families [47], we obtain the following solution:
  (17) $$\theta_{\mathrm{SME}}\left(r\right)=-{\left(E_{r}\left[A\left(x\right)\right]\right)}^{-1}\times \left(E_{r}\left[b\left(x\right)\right]\right),$$
  where
  $$A\left(x\right):={\left[t_{i}^{\prime }\left(x\right)t_{j}^{\prime }\left(x\right)\right]}_{ij}$$
  is a D×D symmetric matrix, and
  $$b\left(x\right)={\left[t_{1}^{\prime \prime }\left(x\right)\ldots \;t_{D}^{\prime \prime }\left(x\right)\right]}^{⊤}$$
  is a *D*-dimensional column vector.

**Theorem 2.**  *The best conversion of a density r(x) into a density $p_{\theta }\left(x\right)$ of an exponential family minimizing the right-sided Hyvärinen divergence is*

$$\theta_{\mathrm{SME}}\left(r\right)=-{\left(E_{r}\left[{\left[t_{i}^{\prime }\left(x\right)t_{j}^{\prime }\left(x\right)\right]}_{ij}\right]\right)}^{-1}\times \left(E_{r}{\left[\left[t_{1}^{\prime \prime }\left(x\right)\ldots \;t_{D}^{\prime \prime }\left(x\right)\right]\right]}^{⊤}\right).$$

- Solution instantiated for polynomial exponential families:
  For polynomial exponential families of order *D*, we have $t_{i}^{\prime }\left(x\right)=ix^{i-1}$ and $t_{i}^{\prime \prime }\left(x\right)=i\left(i-1\right)x^{i-2}$, and therefore, we have
  $$A_{D}=E_{r}\left[A\left(x\right)\right]={\left[ij\;\mu_{i+j-2}\left(r\right)\right]}_{ij},$$
  and
  $$b_{D}=E_{s}\left[b\left(x\right)\right]={\left[j\left(j-1\right)\;\mu_{j-2}\left(r\right)\right]}_{j},$$
  where $\mu_{l}\left(r\right):=E_{r}\left[X^{l}\right]$ denotes the *l*-th raw moment of distribution X∼r(x) (with the convention that $m_{-1}\left(r\right)=0$). For a probability density function r(x), we have $\mu_{1}\left(r\right)=1$.
  Thus, the integral-based SME of a density *r* is:
  (18) $$\theta_{\mathrm{SME}}\left(r\right)=-{\left({\left[ij\mu_{i+j-2}\left(r\right)\right]}_{ij}\right)}^{-1}\times {\left[j\left(j-1\right)\mu_{j-2}\left(r\right)\right]}_{j}.$$
  For example, matrix $A_{4}$ is
  $$\left[\begin{array}{cccc} \mu_{0} & 2\mu_{1} & 3\mu_{2} & 4\mu_{3}\\ 2\mu_{1} & 4\mu_{2} & 6\mu_{3} & 8\mu_{4}\\ 3\mu_{2} & 6\mu_{3} & 9\mu_{4} & 12\mu_{5}\\ 4\mu_{3} & 8\mu_{4} & 12\mu_{5} & 16\mu_{6} \end{array}\right].$$
- Faster PEF solutions using Hankel matrices:
  The method of Cobb et al. [28] (1983) anticipated the Score Matching method of Hyvärinen (2005). It can be derived from Stein’s lemma for exponential families [50]. The integral-based Score Matching method is consistent, i.e., if $r=p_{\theta }$, then ${\bar{\theta }}_{\mathrm{SME}}=\theta$: The probabilistic proof for $r\left(x\right)=p_{e}\left(x\right)$ is reported as Theorem 2 of [28]. The integral-based proof is based on the property that arbitrary order partial mixed derivatives can be obtained from higher-order partial derivatives with respect to $\theta_{1}$ [29]:
  $$\partial_{1}^{i_{1}}\ldots \partial_{D}^{i_{D}}F\left(\theta \right)=\partial_{1}^{\sum_{j=1}^{D}ji_{j}}F\left(\theta \right),$$
  where $\partial_{i}:=\frac{\partial }{\partial \theta_{i}}$.
  The complexity of the direct SME method is $O(D^{3})$ as it requires the inverse of the D×D-dimensional matrix $A_{D}$.
  We show how to lower this complexity by reporting an equivalent method (originally presented in [28]) that relies on recurrence relationships between the moments of $p_{\theta }\left(x\right)$ for PEDs. Recall that $\mu_{l}\left(r\right)$ denotes the *l*-th raw moment $E_{r}\left[x^{l}\right]$.
  Let $A^{\prime }={\left[a_{i+j-2}^{\prime }\right]}_{ij}$ denote the D×D symmetric matrix with $a_{i+j-2}^{\prime }\left(r\right)=\mu_{i+j-2}\left(r\right)$ (with $a_{0}^{\prime }\left(r\right)=\mu_{0}\left(r\right)=1$), and $b^{\prime }={\left[b_{i}\right]}_{i}$ the *D*-dimensional vector with $b_{i}^{\prime }\left(r\right)=\left(i+1\right)\mu_{i}\left(r\right)$. We solve the system $A^{\prime }\beta =b^{\prime }$ to obtain $\beta ={A^{\prime }}^{-1}b^{\prime }$. We then obtain the natural parameter ${\bar{\theta }}_{\mathrm{SME}}$ from the vector β as
  (19) $${\bar{\theta }}_{\mathrm{SME}}=\left[\begin{array}{c} -\frac{\beta_{1}}{2}\\ \vdots \\ -\frac{\beta_{i}}{i+1}\\ \vdots \\ -\frac{\beta_{D}}{D+1} \end{array}\right].$$
  Now, if we inspect matrix $A_{D}^{\prime }=\left[\mu_{i+j-2}\left(r\right)\right]$, we find that matrix $A_{D}^{\prime }$ is a Hankel matrix: A Hankel matrix has constant anti-diagonals and can be inverted in quadratic-time [51,52] instead of cubic time for a general D×D matrix. (The inverse of a Hankel matrix is a Bezoutian matrix [53].) Moreover, a Hankel matrix can be stored using linear memory (store 2D−1 coefficients) instead of quadratic memory of regular matrices.
  For example, matrix $A_{4}^{\prime }$ is:
  $$A_{4}^{\prime }=\left[\begin{array}{cccc} \mu_{0} & \mu_{1} & \mu_{2} & \mu_{3}\\ \mu_{1} & \mu_{2} & \mu_{3} & \mu_{4}\\ \mu_{2} & \mu_{3} & \mu_{4} & \mu_{5}\\ \mu_{3} & \mu_{4} & \mu_{5} & \mu_{6} \end{array}\right],$$
  and requires only 6=2×4−2 coefficients to be stored instead of 4×4=16. The order-*d* moment matrix is
  $$A_{d}^{\prime }:={\left[\mu_{i+j-2}\right]}_{ij}=\left[\begin{array}{cccc} \mu_{0} & \mu_{1} & \ldots & \mu_{d}\\ \mu_{1} & \mu_{2} & \ldots & \vdots \\ \vdots & & \ddots & \vdots \\ \mu_{d} & \ldots & \ldots & \mu_{2d} \end{array}\right],$$
  is a Hankel matrix stored using 2d+1 coefficients:
  $$A_{d}^{\prime }=:\mathrm{Hankel}\left(\mu_{0},\mu_{1},\ldots ,\mu_{2d}\right).$$
  In statistics, those matrices $A_{d}^{\prime }$ are called moment matrices and well-studied [54,55,56]. The variance Var[X] of a random variable *X* can be expressed as the determinant of the order-2 moment matrix:
  $$\mathrm{Var}\left[X\right]=E\left[{\left(X-\mu \right)}^{2}\right]=E\left[X^{2}\right]-E{\left[X\right]}^{2}=\mu_{2}-\mu_{1}^{2}=\det\left(\left[\begin{array}{cc} 1 & \mu_{1}\\ \mu_{1} & \mu_{2} \end{array}\right]\right)\geq 0.$$
  This observation yields a generalization of the notion of variance to d+1 random variables: $X_{1},\ldots ,X_{d+1}{∼}_{iid}F_{X}\Rightarrow E\left[\prod_{j>i}{\left(X_{i}-X_{j}\right)}^{2}\right]=\left(d+1\right)!\;\det\left(M_{d}\right)\geq 0$. The variance can be expressed as $E[\frac{1}{2}{\left(X_{1}-X_{2}\right)}^{2}]$ for $X_{1},X_{2}{∼}_{iid}F_{X}$. See [57] (Chapter 5) for a detailed description related to *U*-statistics.
  For GMMs *r*, the raw moments $\mu_{l}\left(r\right)$ to build matrix $A_{D}$ can be calculated in closed-form, as explained in Section 2.4.

**Theorem 3** (Score matching GMM conversion) *The Score Matching conversion of a GMM m(x) into a polynomial exponential density $p_{\theta_{\mathrm{SME}}\left(m\right)}\left(x\right)$ of order D is obtained as*

$$\theta_{\mathrm{SME}}\left(m\right)=-{\left({\left[ij\;m_{i+j-2}\right]}_{ij}\right)}^{-1}\times {\left[j\left(j-1\right)\;m_{j-2}\right]}_{j},$$

*where $m_{i}=E_{m}\left[x^{i}\right]$ denote the ith non-central moment of the GMM m(x).*

### 2.3. Converting Numerically Moment Parameters from/to Natural Parameters

Recall that our fast heuristic approximates the Jeffreys divergence by

$${\tilde{D}}_{J}\left[m,m^{\prime }\right]:={\left({\bar{\theta }}_{\mathrm{SME}}\left(m^{\prime }\right)-{\bar{\theta }}_{\mathrm{SME}}\left(m\right)\right)}^{⊤}\left({\bar{\eta }}_{\mathrm{MLE}}\left(m^{\prime }\right)-{\bar{\eta }}_{\mathrm{MLE}}\left(m\right)\right).$$

Because *F* and $\nabla F^{*}$ are not available in closed form (except for the case D=2 of the normal family), we cannot obtain θ from a given η (using $\theta =\nabla F^{*}\left(\eta \right)$) nor η from a given θ (using η=∇F(θ)).

However, provided that we can approximate numerically $\tilde{\eta }≃\nabla F\left(\theta \right)$ and $\tilde{\theta }≃\nabla F^{*}\left(\eta \right)$, we also consider these two approximations for the Jeffreys divergence:

$${\tilde{\Delta }}_{J}^{\mathrm{MLE}}\left[m_{1},m_{2}\right]:={\left({\tilde{\theta }}_{2}^{\mathrm{MLE}}-{\tilde{\theta }}_{1}^{\mathrm{MLE}}\right)}^{⊤}\left({\bar{\eta }}_{2}^{\mathrm{MLE}}-{\bar{\eta }}_{1}^{\mathrm{MLE}}\right),$$

and

$${\tilde{\Delta }}_{J}^{\mathrm{SME}}\left[m_{1},m_{2}\right]={\left({\bar{\theta }}_{2}^{\mathrm{SME}}-{\bar{\theta }}_{1}^{\mathrm{SME}}\right)}^{⊤}\left({\tilde{\eta }}_{2}^{\mathrm{SME}}-{\tilde{\eta }}_{1}^{\mathrm{SME}}\right).$$

We show how to numerically estimate ${\tilde{\theta }}^{\mathrm{MLE}}≃\nabla F\left({\bar{\eta }}^{\mathrm{MLE}}\right)$ from ${\bar{\eta }}^{\mathrm{MLE}}$ in Section 2.3.1. Next, in Section 2.3.2, we show how to stochastically estimate ${\tilde{\eta }}^{\mathrm{SME}}≃\nabla F^{*}\left({\bar{\theta }}^{\mathrm{SME}}\right)$.

#### 2.3.1. Converting Moment Parameters to Natural Parameters Using Maximum Entropy

Let us report the iterative approximation technique of [58] (which extended the method described in [35]) based on solving a maximum entropy problem (MaxEnt problem). This method will be useful when comparing our fast heuristic ${\tilde{D}}_{J}\left[m,m^{\prime }\right]$ with the approximations ${\tilde{\Delta }}_{J}^{\mathrm{MLE}}\left[m,m^{\prime }\right]$ and ${\tilde{\Delta }}_{J}^{\mathrm{SME}}\left[m,m^{\prime }\right]$.

The density $p_{\theta }$ of any exponential family can be characterized as a maximum entropy distribution given the *D* moment constraints $E_{p_{\theta }}\left[t_{i}\left(x\right)\right]=\eta_{i}$: Namely, $\max_{p}h\left(p\right)$ subject to the D+1 moment constraints $\int t_{i}\left(x\right)p\left(x\right)dx=\eta_{i}$ for i∈{0,…,D}, where we added by convention $\eta_{0}=1$ and $t_{0}\left(x\right)=1$ (so that ∫p(x)dx=1). The solution of this MaxEnt problem [58] is $p\left(x\right)=p_{\lambda }$, where λ are the D+1 Lagrangian parameters. Here, we adopt the following canonical parameterization of the densities of an exponential family:

$$p_{\lambda }\left(x\right):=\exp\left(-\sum_{i=0}^{D}\lambda_{i}t_{i}\left(x\right)\right).$$

That is, $F\left(\lambda \right)=\lambda_{0}$ and $\lambda_{i}=-\theta_{i}$ for i∈{1,…,D}. Parameter λ is a kind of augmented natural parameter that includes the log-normalizer in its first coefficient.

Let $K_{i}\left(\lambda \right):=E_{p_{\theta }}\left[t_{i}\left(x\right)\right]=\eta_{i}$ denote the set of D+1 non-linear equations for i∈{0,…,D}. The Iterative Linear System Method [58] (ILSM) converts $p^{\eta }$ to $p_{\theta }$ iteratively. We initialize $\lambda^{\left(0\right)}$ to ${\bar{\theta }}_{\mathrm{SME}}$ (and calculate numerically $\lambda_{0}^{\left(0\right)}=F\left({\bar{\theta }}_{\mathrm{SME}}\right)$).

At iteration *t* with current estimate $\lambda^{\left(t\right)}$, we use the following first-order Taylor approximation:

$$K_{i}\left(\lambda \right)\approx K_{i}\left(\lambda^{\left(t\right)}\right)+\left(\lambda -\lambda^{\left(t\right)}\right)\nabla K_{i}\left(\lambda^{\left(t\right)}\right).$$

Let H(λ) denote the (D+1)×(D+1) matrix:

$$H\left(\lambda \right):={\left[\frac{\partial K_{i}\left(\lambda \right)}{\partial \theta_{j}}\right]}_{ij}.$$

We have

$$H_{ij}\left(\lambda \right)=H_{ji}\left(\lambda \right)=-E_{p_{\theta }}\left[t_{i}\left(x\right)t_{j}\left(x\right)\right].$$

We update as follows:

(20) $$\lambda^{\left(t+1\right)}=\lambda^{\left(t\right)}+H^{-1}\left(\lambda^{\left(t\right)}\right)\left[\begin{array}{c} \eta_{0}-K_{0}\left(\lambda^{\left(t\right)}\right)\\ \vdots \\ \eta_{D}-K_{D}\left(\lambda^{\left(t\right)}\right) \end{array}\right].$$

For a PEF of order *D*, we have

$$H_{ij}\left(\lambda \right)=-E_{p_{\theta }}\left[x^{i+j-2}\right]=-\mu_{i+j-2}\left(p_{\theta }\right).$$

This yields a moment matrix $H_{\lambda }$ (Hankel matrix), which can be inverted in quadratic time [52]. In our setting, the moment matrix is invertible because |H|>0, see [59].

Let ${\tilde{\lambda }}_{T}\left(\eta \right)$ denote $\theta^{\left(T\right)}$ after *T* iterations (retrieved from $\lambda^{\left(T\right)}$) and the corresponding natural parameter of the PED. We have the following approximation of the JD:

$$D_{J}\left[m,m^{\prime }\right]\approx {\left({\tilde{\theta }}_{T}\left(\eta^{\prime }\right)-{\tilde{\theta }}_{T}\left(\eta \right)\right)}^{⊤}\left(\eta^{\prime }-\eta \right).$$

The method is costly because we need to numerically calculate $\mu_{i+j-2}\left(p_{\theta }\right)$ and the $K_{i}$’s (e.g., univariate Simpson integrator). Another potential method consists of estimating these expectations using acceptance-rejection sampling [60,61]. We may also consider the holonomic gradient descent [29]. Thus, the conversion η→θ method is costly. Our heuristic ${\tilde{\Delta }}_{J}$ bypasses this costly moment-to-natural parameter conversion by converting each mixture *m* to a pair $\left(p_{\theta_{\mathrm{SME}}},p_{\eta_{\mathrm{MLE}}}\right)$ of PEDs parameterized in the natural and moment parameters (i.e., loosely speaking, we untangle these dual parameterizations).

#### 2.3.2. Converting Natural Parameters to Moment Parameters

Given a PED $p_{\theta }\left(x\right)$, we have to find its corresponding moment parameter η (i.e., $p_{\theta }=p^{\eta }$). Since $\eta =E_{p_{\theta }}\left[t\left(x\right)\right]$, we sample *s* i.i.d. variates $x_{1},\ldots ,x_{s}$ from $p_{\theta }$ using acceptance-rejection sampling [60,61] or any other Markov chain Monte Carlo technique [62] and estimate $\hat{\eta }$ as:

$$\hat{\eta }=\frac{1}{s}\sum_{i=1}^{s}t\left(x_{i}\right).$$

### 2.4. Raw Non-Central Moments of Normal Distributions and GMMs

In order to implement the MLE or SME Gaussian mixture conversion procedures, we need to calculate the raw moments of a Gaussian mixture model. The *l*-th moment raw moment $E[Z^{l}]$ of a standard normal distribution Z∼N(0,1) is 0 when *l* is odd (since the normal standard density is an even function) and $\left(l-1\right)!!=2^{-\frac{l}{2}}\frac{l!}{(l/2)!}$ when *l* is even, where $n!!=\sqrt{\frac{2^{n+1}}{\pi }}\Gamma \left(\frac{n}{2}+1\right)=\prod_{k=0}^{\lceil \frac{n}{2}\rceil -1}\left(n-2k\right)$ is the double factorial (with (−1)!!=1 by convention). Using the binomial theorem, we deduce that a normal distribution X=μ+σZ has finite moments:

μl(pμ,σ)=Epμ,σ[Xl]=E[(μ+σZ)l]=E[(μ+σZ)l]=∑i=0lliμl−iσiE[Zi].

That is, we have

(21) μl(pμ,σ)=∑i=0⌊l2⌋li(2i−1)!!μl−2iσ2i,

where n!! denotes the double factorial:

$$n!!=\prod_{k=0}^{\left\lceil \frac{n}{2}\right]-1}\left(n-2k\right)=\left\{\begin{array}{cc} \prod_{k=1}^{\frac{n}{2}}\left(2k\right) & n\;\mathrm{is}\;\mathrm{even},\\ \prod_{k=1}^{\frac{n+1}{2}}\left(2k-1\right) & n\;\mathrm{is}\;\mathrm{odd}. \end{array}\right.$$

By the linearity of the expectation E[·], we deduce the *l*-th raw moment of a GMM $m\left(x\right)=\sum_{i=1}^{k}w_{i}p_{\mu_{i},\sigma_{i}}\left(x\right)$:

$$\mu_{l}\left(m\right)=\sum_{i=1}^{k}w_{i}\mu_{l}\left(p_{\mu_{I},\sigma_{i}}\right).$$

Notice that by using [63], we can extend this formula to truncated normals and GMMs. Thus, computing the first O(D) raw moments of a GMM with *k* components can be done in $O(kD^{2})$ using the Pascal triangle method for computing the binomial coefficients. See also [64].

## 3. Goodness-of-Fit between GMMs and PEDs: Higher Order Hyvärinen Divergences

Once we have converted a GMM m(x) into an unnormalized PED $q_{\theta_{m}}\left(x\right)={\tilde{p}}_{\theta_{m}}\left(x\right)$, we would like to evaluate the quality of the conversion, i.e., $D[m\left(x\right):q_{\theta_{m}}\left(x\right)]$, using a statistical divergence D[·:·]. This divergence shall allow us to perform model selection by choosing the order *D* of the PEF so that $D[m\left(x\right):p_{\theta }\left(x\right)]\leq \epsilon$ for $\theta \in R^{D}$, where ϵ>0 is a prescribed threshold. Since PEDs have computationally intractable normalization constants, we consider a right-sided projective divergence [42] D[p:q] that satisfies $D\left[p:\lambda q\right]=D\left[p:q\right]=D\left[p:\tilde{q}\right]$ for any λ>0. For example, we may consider the γ-divergence [65] that is a two-sided projective divergence: $D_{\gamma }\left[\lambda p:\lambda^{\prime }q\right]=D\left[p:q\right]=D\left[\tilde{p}:\tilde{q}\right]$ for any $\lambda ,\lambda^{\prime }>0$ and converge to the KLD when γ→0. However, the γ-divergence between a mixture model and an unnormalized PEF does not yield a closed-form formula. Moreover, the γ-divergence between two unnormalized PEDs is expressed using the log-normalizer function F(·) that is computationally intractable [66].

In order to a get a closed-form formula for a divergence between a mixture model and an unnormalized PED, we consider the order-α (for α>0) Hyvärinen divergence [42] as follows:

(22) $$D_{H,\alpha }\left[p:q\right]:=\int p{\left(x\right)}^{\alpha }\;{\left(\nabla_{x}\log p\left(x\right)-\nabla_{x}\log q\left(x\right)\right)}^{2}dx,\;\alpha >0.$$

The Hyvärinen divergence [42] (order-1 Hyvärinen divergence) has also been called the Fisher divergence [27,67,68,69] or relative Fisher information [48]. Notice that when α=1, $D_{H,1}\left[p:q\right]=D_{H}\left[p:q\right]$, the ordinary Hyvärinen divergence [27].

The Hyvärinen divergences $D_{H,\alpha }$ is a right-sided projective divergence, meaning that the divergence satisfies $D_{H,\alpha }\left[p:q\right]=D_{H,\alpha }\left[p:\lambda q\right]$ for any λ>0. That is, we have $D_{H,\alpha }\left[p:q\right]=D_{H,\alpha }\left[p:\tilde{q}\right]$. Thus, we have $D_{H,\alpha }\left[m:p_{\theta }\right]=D_{H,\alpha }\left[m:q_{\theta }\right]$ for an unnormalized PED $q_{\theta }={\tilde{p}}_{\theta }$. For statistical estimation, it is enough to have a sided projective divergence since we need to evaluate the goodness of fit between the (normalized) empirical distribution $p_{e}$ and the (unnormalized) parameteric density.

For univariate distributions, $\nabla_{x}\log p\left(x\right)=\frac{p^{\prime }\left(x\right)}{p(x)}$, and $\frac{p^{\prime }\left(x\right)}{p(x)}=\frac{{\tilde{p}}^{\prime }\left(x\right)}{\tilde{p}\left(x\right)}$, where $\tilde{p}\left(x\right)$ is the unnormalized model.

Let $P_{\theta }\left(x\right):=\sum_{i=1}^{D}\theta_{i}x^{i}$ be a homogeneous polynomial defining the shape of the EPF:

$$p_{\theta }\left(x\right)=\exp\left(P_{\theta }\left(x\right)-F\left(\theta \right)\right).$$

For PEDs with the homogeneous polynomial $P_{\theta }\left(x\right)$, we have $\frac{p^{\prime }\left(x\right)}{p(x)}={\left(\log P_{\theta }\left(x\right)\right)}^{\prime }=\sum_{i=1}^{D}i\theta_{i}x^{i-1}$.

**Theorem** **4.** *The Hyvärinen divergence $D_{H,2}\left[m:q_{\theta }\right]$ of order 2 between a Gaussian mixture m(x) and a polynomial exponential family density $q_{\theta }\left(x\right)$ is available in closed form.*

**Proof.**  We have $D_{H,2}\left[m:q\right]=\int m{\left(x\right)}^{2}\;{\left(\frac{m^{\prime }\left(x\right)}{m(x)}-\sum_{i=1}^{D}i\theta_{i}x^{i-1}\right)}^{2}dx$ with

$$m^{\prime }\left(x\right)=-\sum_{i=1}^{k}w_{i}\frac{x-\mu_{i}}{\sigma_{i}^{2}}p\left(x_{i};\mu_{i},\sigma_{i}\right),$$

denoting the derivative of the Gaussian mixture density m(x). It follows that:

$$D_{H,2}\left[m:q\right]=\int m^{\prime }\left(x\right)dx-2\sum_{i=1}^{D}i\theta_{i}\int x^{i-1}m^{\prime }\left(x\right)m\left(x\right)dx+\sum_{i,j=1}^{D}ij\theta_{i}\theta_{j}\int x^{i+j-2}m{\left(x\right)}^{2}dx,$$

where

$$\int x^{i}m^{\prime }\left(x\right)m\left(x\right)dx=-\sum w_{a}w_{b}\int \frac{x-\mu_{a}}{\sigma_{a}^{2}}x^{i}p\left(x;\mu_{a},\sigma_{a}\right)p\left(x;\mu_{b},\sigma_{b}\right)dx.$$

Therefore, we have

$$D_{H,2}\left[m:q\right]=\int m^{\prime }\left(x\right)dx-2\sum_{i=1}^{D}i\theta_{i}\int x^{i-1}m^{\prime }\left(x\right)m\left(x\right)dx+\sum_{i,j=1}^{D}ij\theta_{i}\theta_{j}\int x^{i+j-2}m{\left(x\right)}^{2}dx$$

with $m^{\prime }\left(x\right)=-\sum w_{a}\frac{x-\mu_{a}}{\sigma_{a}^{2}}p\left(x;\mu_{a},\sigma_{a}\right)$.

Since $p_{a}\left(x\right)p_{b}\left(x\right)=\kappa_{a,b}p\left(x;\mu_{ab},\sigma_{ab}\right)$, with

$$\begin{array}{ccc} \mu_{ab} & = & \sigma_{a}^{2}\sigma_{b}^{2}\left(\sigma_{b}^{2}\mu_{a}+\sigma_{a}^{2}\mu_{b}\right),\\ \sigma_{ab} & = & \frac{\sigma_{a}\sigma_{b}}{\sqrt{\sigma_{a}^{2}+\sigma_{b}^{2}}},\\ \kappa_{a,b} & = & \exp(F\left(\mu_{ab},\sigma_{ab}\right)-F\left(\mu_{a},\sigma_{a}\right)-F\left(\mu_{b},\sigma_{b}\right)), \end{array}$$

and

$$F\left(\mu ,\sigma \right)=\frac{\mu^{2}}{2\sigma^{2}}+\frac{1}{2}\log\left(2\pi \sigma^{2}\right),$$

the log-normalizer of the Gaussian exponential family [42].

Therefore, we obtain

$$\int p_{a}\left(x\right)p_{b}\left(x\right)x^{l}dx=\kappa_{a,b}m_{l}\left(\mu_{ab},\sigma_{ab}\right).$$

Thus, the Hyvärinen divergence $D_{H,2}$ of order 2 between a GMM and a PED is available in closed-form. □

For example, when k=1 (i.e., mixture *m* is a single Gaussian $p_{\mu_{1},\sigma_{1}}$) and $p_{\theta }$ is a normal distribution (i.e., PED with D=2, $q_{\theta }=p_{\mu_{2},\sigma_{2}}$), we obtain the following formula for the order-2 Hyvärinen divergence:

$$D_{H,2}\left[p_{\mu_{1},\sigma_{1}}:p_{\mu_{2},\sigma_{2}}\right]=\frac{{\left(\sigma_{1}^{2}-\sigma_{2}^{2}\right)}^{2}+2{\left(\mu_{2}-\mu_{1}\right)}^{2}\sigma_{1}^{2}}{8\sqrt{\pi }\sigma_{1}^{3}\sigma_{2}^{4}}.$$

## 4. Experiments: Jeffreys Divergence between Mixtures

In this section, we evaluate our heuristic to approximate the Jeffreys divergence between two mixtures *m* and $m^{\prime }$:

$${\tilde{D}}_{J}\left[m,m^{\prime }\right]:={\left({\bar{\theta }}_{\mathrm{SME}}\left(m^{\prime }\right)-{\bar{\theta }}_{\mathrm{SME}}\left(m\right)\right)}^{⊤}\left({\bar{\eta }}_{\mathrm{MLE}}\left(m^{\prime }\right)-{\bar{\eta }}_{\mathrm{MLE}}\left(m\right)\right).$$

Recall that stochastically estimating the JD between *k*-GMMs with Monte Carlo sampling using *s* samples (i.e., ${\hat{D}}_{J,s}\left[m:m^{\prime }\right]$) requires $\tilde{O}\left(ks\right)$ and is not deterministic. That is, different MC runs yield fluctuating values that may be fairly different. In comparison, approximating $D_{J}$ by ${\tilde{D}}_{J}$ using $\Delta_{J}$ by converting mixtures to *D*-order PEDs require $O(kD^{2})$ time to compute the raw moments and $O(D^{2})$ time to invert a Hankel moment matrix. Thus, by choosing D=2k, we obtain a deterministic $O(k^{3})$ algorithm that is faster than the MC sampling when $k^{2}\ll s$. Since there are, at most, *k* modes for a *k*-GMM, we choose order D=2k for the PEDs.

To obtain quantitative results on the performance of our heuristic ${\tilde{D}}_{J}$, we build random GMMs with *k* components as follows: $m\left(x\right)=\sum_{i=1}^{k}w_{i}p_{\mu_{i},\sigma_{i}}\left(x\right)$, where $w_{i}∼U_{i}$, $\mu_{i}∼-10+10U_{1}^{\prime }$ and $\sigma_{i}∼1+U_{2}^{\prime }$, where the $U_{i}$’s, and $U_{1}^{\prime }$ and $U_{2}^{\prime }$ are independent uniform distributions on [0,1). The mixture weights are then normalized to sum up to one. For each value of *k*, we make 1000 trial experiments to gather statistics and use $s=10^{5}$ for evaluating the Jeffreys divergence ${\hat{D}}_{J}$ by Monte Carlo samplings. We denote by $\mathrm{error}:=\frac{|{\hat{D}}_{J}-\Delta_{J}|}{{\hat{D}}_{J}}$ the error of an experiment. Table 1 presents the results of the experiments for D=2k: The table displays the average error, the maximum error (minimum error is very close to zero, of order $10^{-5}$), and the speed-up obtained by our heuristic $\Delta_{J}$. Those experiments were carried out on a Dell Inspiron 7472 laptop (equipped with an Intel(R) Core(TM) i5-8250U CPU at 1.60 GHz).

Notice that the quality of the approximations of ${\tilde{D}}_{J}$ depend on the number of modes of the GMMs. However, calculating the number of modes is difficult [43,70], even for simple cases [71,72].

**Figure 4:**
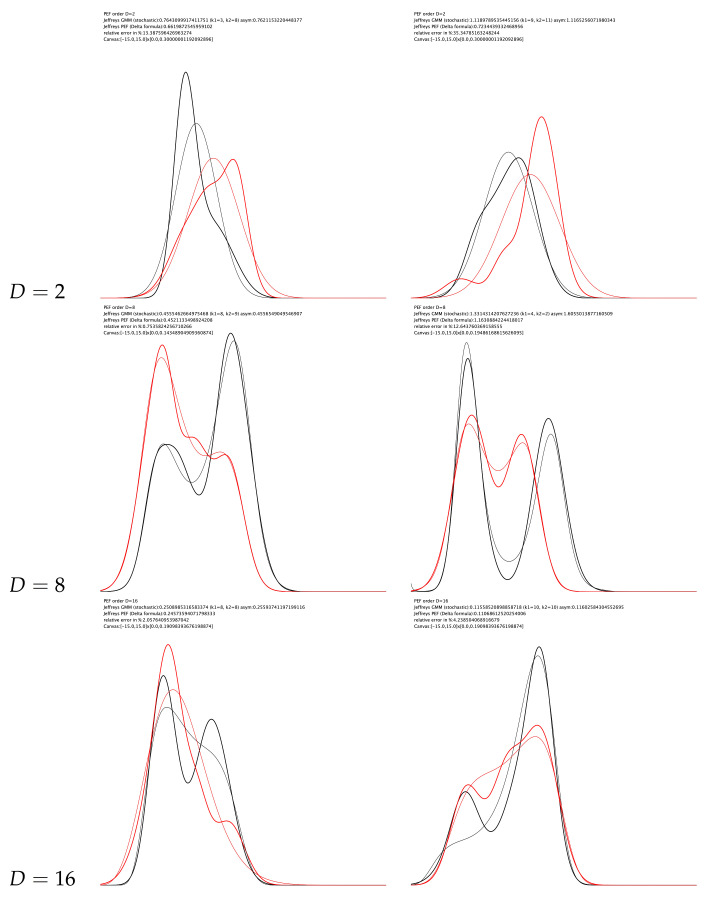
Experiments of approximating the Jeffreys divergence between two mixtures by considering pairs of PEDs. Notice that only the PEDs estimated using the Score Matching in the natural parameter space are displayed.

Figure 4 displays several experiments of converting mixtures to pairs of PEDs to obtain approximations of the Jeffreys divergence.

**Figure 5:**
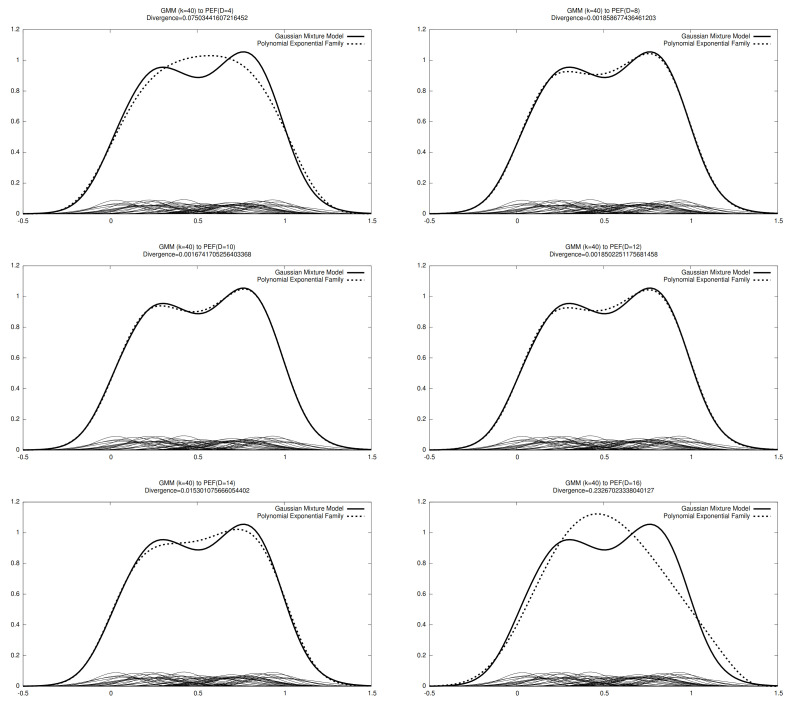
Selecting the PED order *D* my evaluating the best divergence order-2 Hyvärinen divergence (for D∈{4,8,10,12,14,16}) values. Here, the order D=10 (boxed) yields the lowest order-2 Hyvärinen divergence: The GMM is close to the PED.

Figure 5 illustrates the use of the order-2 Hyvärinen divergence $D_{H,2}$ to perform model selection for choosing the order of a PED.

**Figure 6:**
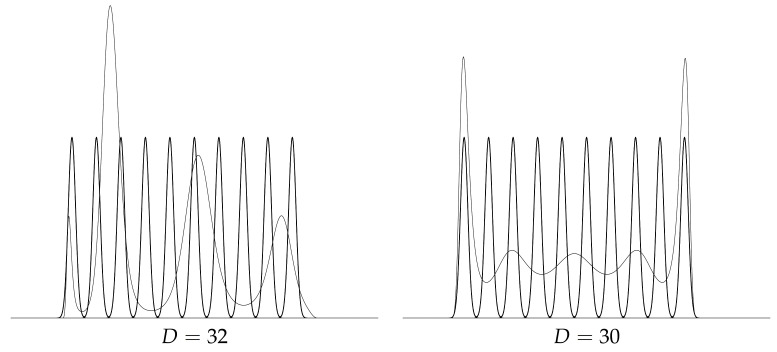
Some limitation examples of the conversion of GMMs (black) to PEDs (grey) using the integral-based Score Matching estimator: Case of GMMs with many modes.

Finally, Figure 6 displays some limitations of the GMM to PED conversion when the GMMs have many modes. In that case, running the conversion ${\bar{\eta }}_{\mathrm{MLE}}$ to obtain ${\tilde{\theta }}_{T}\left({\bar{\eta }}_{\mathrm{MLE}}\right)$ and estimate the Jeffreys divergence by

$${\tilde{\Delta }}_{J}^{\mathrm{MLE}}\left[m_{1},m_{2}\right]={\left({\tilde{\theta }}_{2}^{\mathrm{MLE}}-{\tilde{\theta }}_{1}^{\mathrm{MLE}}\right)}^{⊤}\left({\bar{\eta }}_{2}^{\mathrm{MLE}}-{\bar{\eta }}_{1}^{\mathrm{MLE}}\right),$$

improves the results but requires more computation.

**Figure 7:**
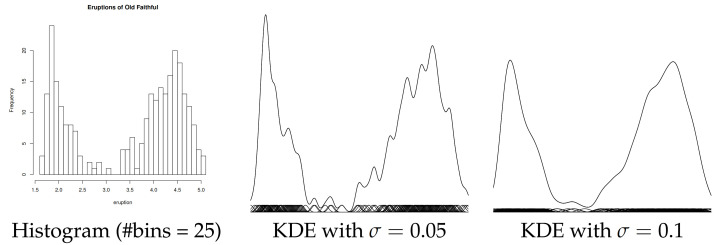
Modeling the Old Faithful geyser by a KDE (GMM with k=272 components, uniform weights $w_{i}=\frac{1}{272}$): Histogram (#bins = 25) (**left**), KDE with σ=0.05 (**middle**), and KDE with σ=0.1 with less spurious bumps (**right**).

**Figure 8:**
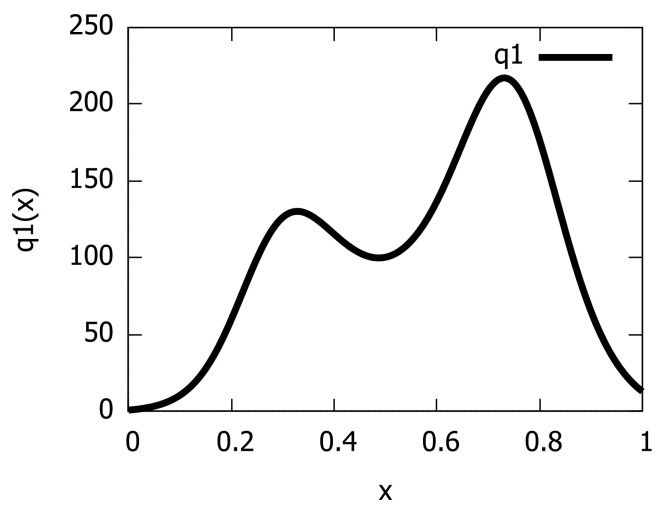
Modeling the Old Faithful geyser by an exponential-polynomial distribution of order D=10.

Next, we consider learning a PED by converting a GMM derived itself from a Kernel Density Estimator (KDE). We use the duration of the eruption for the Old Faithful geyser in Yellowstone National Park (Wyoming, USA): The dataset consists of 272 observations (https://www.stat.cmu.edu/~larry/all-of-statistics/=data/faithful.dat) (access date: 25 October 2021) and is included in the R language package ‘stats’. Figure 7 displays the GMMs obtained from the KDEs of the Old Faithful geyser dataset when choosing for each component σ=0.05 (left) and σ=0.1. Observe that the data are bimodal once the spurious modes (i.e., small bumps) are removed, as studied in [32]. Barron and Sheu [32] modeled that dataset using a bimodal PED of order D=4, i.e., a quartic distribution. We model it with a PED of order D=10 using the integral-based Score Matching method. Figure 8 displays the unnormalized bimodal density $q_{1}$ (i.e., ${\tilde{p}}_{1}$) that we obtained using the integral-based Score Matching method (with X=(0,1)).

## 5. Conclusions and Perspectives

Many applications [7,73,74,75] require computing the Jeffreys divergence (the arithmetic symmetrization of the Kullback–Leibler divergence) between Gaussian mixture models. Since the Jeffreys divergence between GMMs is provably not available in closed-form [13], one often ends up implementing a costly Monte Carlo stochastic approximation of the Jeffreys divergence. In this paper, we first noticed the simple expression of the Jeffreys divergence between densities $p_{\theta }$ and $p_{\theta^{\prime }}$ of an exponential family using their dual natural and moment parameterizations [22] $p_{\theta }=p^{\eta }$ and $p_{\theta^{\prime }}=p^{\eta^{\prime }}$:

$$D_{J}\left[p_{\theta },p_{\theta^{\prime }}\right]={\left(\theta^{\prime }-\theta \right)}^{⊤}\left(\eta^{\prime }-\eta \right),$$

where η=∇F(θ) and $\eta^{\prime }=\nabla F\left(\theta^{\prime }\right)$ for the cumulant function F(θ) of the exponential family. This led us to propose a simple and fast heuristic to approximate the Jeffreys divergence between Gaussian mixture models: First, convert a mixture *m* to a pair $\left(p_{{\bar{\theta }}^{\mathrm{SME}}},p^{{\bar{\eta }}^{\mathrm{MLE}}}\right)$ of dually parameterized polynomial exponential densities using extensions of the Maximum Likelihood and Score Matching Estimators (Theorems 1 and 3), and then approximate the JD deterministically by

$$D_{J}\left[m_{1},m_{2}\right]≃{\tilde{D}}_{J}\left[m_{1},m_{2}\right]={\left({\tilde{\theta }}_{2}^{\mathrm{MLE}}-{\tilde{\theta }}_{1}^{\mathrm{MLE}}\right)}^{⊤}\left({\bar{\eta }}_{2}^{\mathrm{MLE}}-{\bar{\eta }}_{1}^{\mathrm{MLE}}\right).$$

**Figure 9:**
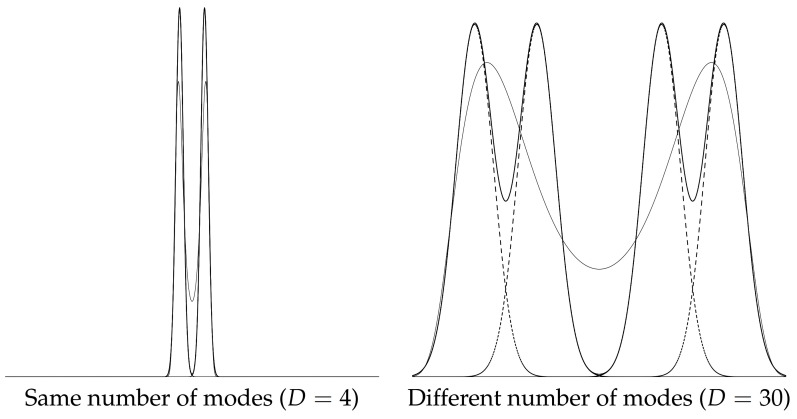
GMM modes versus PED modes: (**left**) same number and locations of modes for the GMM and the PED; (**right**) 4 modes for the GMM but only 2 modes for the PED.

The order of the polynomial exponential family may be either prescribed or selected using the order-2 Hyvärinen divergence, which evaluates in closed form the dissimilarity between a GMM and a density of an exponential-polynomial family (Theorem 4). We experimentally demonstrated that the Jeffreys divergence between GMMs can be reasonably well approximated by ${\tilde{D}}_{J}$ for mixtures with a small number of modes, and we obtained an overall speed-up of several order of magnitudes compared to the Monte Carlo sampling method. We also propose another deterministic heuristic to estimate $D_{J}$ as

$${\tilde{D}}_{J}^{\mathrm{MLE}}\left[m_{1}:m_{2}\right]={\left({\tilde{\theta }}_{2}^{\mathrm{MLE}}-{\tilde{\theta }}_{1}^{\mathrm{MLE}}\right)}^{⊤}\left({\bar{\eta }}_{2}^{\mathrm{MLE}}-{\bar{\eta }}_{1}^{\mathrm{MLE}}\right),$$

where ${\tilde{\theta }}^{\mathrm{MLE}}\approx \nabla F\left({\bar{\eta }}^{\mathrm{MLE}}\right)$ is numerically calculated using an iterative conversion procedure based on maximum entropy [58] (Section 2.3.1). Our technique extends to other univariate mixtures of exponential families (e.g., mixtures of Rayleigh distributions, mixtures of Gamma distributions, or mixtures of Beta distributions, etc). One limitation of our method is that the PED modeling of a GMM may not guarantee obtaining the same number of modes as the GMM even when we increase the order *D* of the exponential-polynomial densities. This case is illustrated in Figure 9 (right).

Although PEDs are well-suited to calculate Jeffreys divergence compared to GMMs, we point out that GMMs are better suited for sampling, while PEDs require Monte Carlo methods (e.g., adaptive rejection sampling or MCMC methods [62]). Furthermore, we can estimate the Kullback–Leibler divergence between two PEDs using rejection sampling (or other McMC methods [62]) or by using the γ-divergence [76] with γ close to zero [66] (e.g., γ=0.001). The web page of the project is https://franknielsen.github.io/JeffreysDivergenceGMMPEF/index.html (accessed on 25 October 2021).

This work opens up several perspectives for future research: For example, we may consider bivariate polynomial-exponential densities for modeling bivariate Gaussian mixture models [29], or we may consider truncating the GMMs in order to avoid tail phenomena when converting GMMs to PEDs [77,78].

## Figures and Tables

**Table 1:** Comparison of ${\tilde{\Delta }}_{J}\left(m_{1},m_{2}\right)$ with ${\hat{D}}_{J}\left(m_{1},m_{2}\right)$ for random GMMs.

*k*	*D*	Average Error	Maximum Error	Speed-Up
2	4	0.1180799978221536	0.9491425404132259	2008.2323536011806
3	6	0.12533811294546526	1.9420608151988419	1010.4917042114389
4	8	0.10198448868508087	5.290871019594698	474.5135294829539
5	10	0.06336388579897352	3.8096955246161848	246.38780782640987
6	12	0.07145257192133717	1.0125283726458822	141.39097909641052
7	14	0.10538875853178625	0.8661463142793943	88.62985036546912
8	16	0.4150905507007969	0.4150905507007969	58.72277575395611

## Data Availability

Not applicable.
